# The cellular protrusions for inter-cellular material transfer: similarities between filopodia, cytonemes, tunneling nanotubes, viruses, and extracellular vesicles

**DOI:** 10.3389/fcell.2024.1422227

**Published:** 2024-07-05

**Authors:** Hooi Ting Hu, Tamako Nishimura, Hiroki Kawana, Rachelle Anne So Dante, Gisela D’Angelo, Shiro Suetsugu

**Affiliations:** ^1^ Division of Biological Science, Graduate School of Science and Technology, Nara Institute of Science and Technology, Nara, Japan; ^2^ Institut Curie, PSL Research University, Centre national de la recherche scientifique (CNRS), Paris, France; ^3^ Data Science Center, Nara Institute of Science and Technology, Nara, Japan; ^4^ Center for Digital Green-innovation, Nara Institute of Science and Technology, Nara, Japan

**Keywords:** filopodia, cytoneme, microvilli, cilia, nanotube tunneling, virus, cancer, platelet

## Abstract

Extracellular vesicles (EVs) are crucial for transferring bioactive materials between cells and play vital roles in both health and diseases. Cellular protrusions, including filopodia and microvilli, are generated by the bending of the plasma membrane and are considered to be rigid structures facilitating various cellular functions, such as cell migration, adhesion, and environment sensing. Compelling evidence suggests that these protrusions are dynamic and flexible structures that can serve as sources of a new class of EVs, highlighting the unique role they play in intercellular material transfer. Cytonemes are specialized filopodia protrusions that make direct contact with neighboring cells, mediating the transfer of bioactive materials between cells through their tips. In some cases, these tips fuse with the plasma membrane of neighboring cells, creating tunneling nanotubes that directly connect the cytosols of the adjacent cells. Additionally, virus particles can be released from infected cells through small bud-like of plasma membrane protrusions. These different types of protrusions, which can transfer bioactive materials, share common protein components, including I-BAR domain-containing proteins, actin cytoskeleton, and their regulatory proteins. The dynamic and flexible nature of these protrusions highlights their importance in cellular communication and material transfer within the body, including development, cancer progression, and other diseases.

## 1 Introduction

**FIGURE 1:**
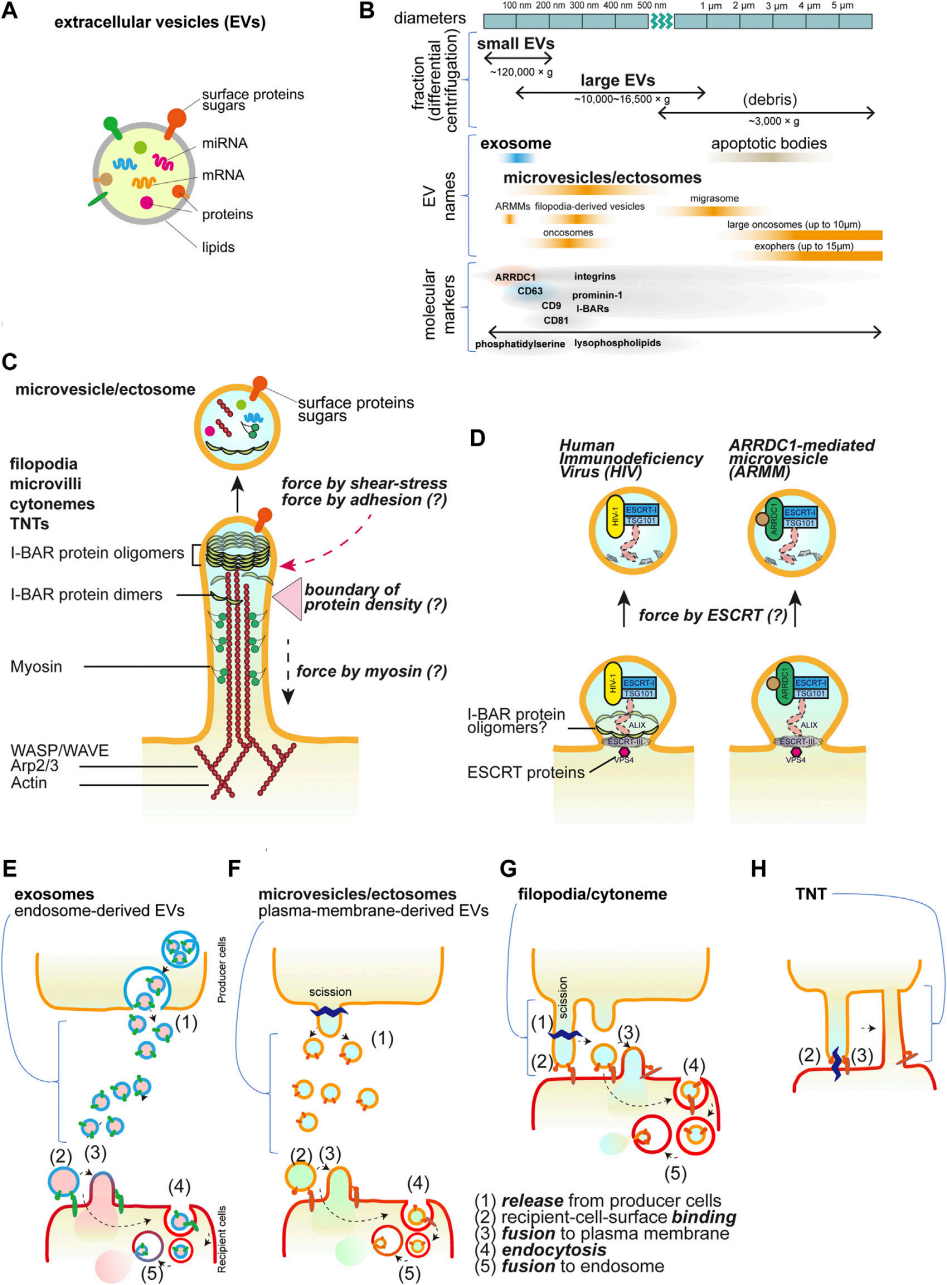
Extracellular vesicles and cellular protrusions. **(A)** The schematic illustration of the extracellular vesicles. **(B)** The EV nomenclature in fractionation and the others. The approximate sizes in diameter are shown with the names in the centrifugal separation of EVs as well as the commonly used names, i.e., exosomes and microvesicles/ectosomes. The names dependent on the origins are also shown. ARRDC1-mediated microvesicles (ARRMs) (Nabhan et al., 2012), filopodia-derived vesicles (FDVs) (Nishimura et al., 2021), migrasomes from the cellular retraction fibers (Ma et al., 2015), oncosomes from cancer cells (Meehan et al., 2016), exophers that contain the subcellular organelles and protein aggregates (Melentijevic et al., 2017), and apoptotic bodies that are the fragments of the apoptotic cells (Kakarla et al., 2020). The representative proteins that are analyzed for EVs are shown, though there is incomplete correspondence to the fractionation and the names. **(C)** The core proteins of the cellular protrusions of filopodia, cytonemes, microvilli, and tunneling nanotubes (TNTs). The actin filaments and motor protein myosins on the filaments, their regulators, and the I-BAR proteins are the key core proteins. The protrusions can release the vesicles by cutting, which occurs by the friction force of the shear flow and presumably by the pulling force associated with the adhesion. The differential localization of the core proteins will make a boundary for cutting. **(D)** Small buds of the plasma membrane for vesicle release. The HIV and ARMM are generated by the ESCRT protein complex to mediate the cutting. The I-BAR protein is involved in HIV release. **(E-H)** Schematics of transfer of the bioactive material inside vesicles or by TNTs. Vesicles are released and then bind to the recipient cells, followed by the fusion with the plasma membrane or by the endocytosis of the vesicles. The endocytosed vesicles are then fused to the endosomal membrane to release the materials inside the vesicles. Each step is marked by the numbers: (1) release from producer cells, (2) recipient-cell-surface binding, (3) fusion to plasma membrane, (4) endocytosis, and (5) fusion to endosome. **(E)** Exosomes, **(F)** microvesicles or ectosomes, **(G)** filopodia/cytonemes, which are the same as **(F)**, but the vesicles are generated after binding to the recipient cells, i.e., no free travel of vesicles occurs, and **(H)** TNTs, which have the tip of the protrusions fused to the recipient cells, facilitate the connection of the cytoplasm.

Extracellular vesicles (EVs) play a crucial role in exchanging bioactive materials, such as proteins, nucleic acids, metabolites, and lipids, between cells (Thery et al., 2018) (Figure 1A). These EVs are naturally secreted by most cells and can be found in various biological fluids, including blood, urine, saliva, cerebrospinal fluid, amniotic fluid, and seminal fluid (Yáñez-Mó et al., 2015). As studies on EVs expand under various conditions, different nomenclatures are used depending on different experimental models, molecular markers, biological conditions, and discovered roles. Efforts are being made to standardize terminology among researchers working in the field of EVs (Welsh et al., 2024). The exact process of EV biogenesis is not completely understood, but one major source of EVs is endosomes, which are the membrane organelles for material transport to/from the plasma membrane and to lysosomes. Intraluminal vesicles (ILVs) in endosomes are secreted by the fusion of endosomes with the plasma membrane. While the term “exosomes” is often used interchangeably with EVs, exosomes specifically refer to EVs derived from endosomes (Welsh et al., 2024). On the other hand, EVs can also be derived directly from the plasma membrane and are known as microvesicles or ectosomes (D’Angelo et al., 2023; Rilla, 2021). Importantly, the name, microvesicles, implies that these microvesicles might be larger than the endosome-derived EVs or exosomes. However, there are overlaps in their sizes, and they cannot be completely separated using ultra-centrifugal separation, which gives the “small EV” and “large EV” fractions (Hu et al., 2021; Welsh et al., 2024) (Figure 1B). Cells continuously reshape their plasma membrane, extending various kinds of membrane protrusions (Chhabra and Higgs, 2007). Membrane protrusions were traditionally considered to be stiff and rigid structures used for cell migration and adhesion, serving as substrate anchors. However, emerging studies suggest that some cellular protrusions are dynamic and flexible, can exist in diverse forms, and can be transformed into EVs (D’Angelo et al., 2023; Rilla, 2021).

In this review, we will discuss the similarities between different types of plasma membrane protrusions, including filopodia, cytoneme, microvilli, tunneling nanotubes (TNTs), cilia, pseudopods (pro platelets) from megakaryocytes, and small protrusions of viral budding, and their role as sources of EVs.

## 2 The building blocks of the protrusions

### 2.1 Cytoplasmic proteins

Most membrane protrusions, including filopodia at the cell periphery and microvilli at the apical surface of cells, share similar proteins (Figure 1C). Actin filaments are the cellular cytoskeleton that provides mechanical stuffiness. The actin filaments are generated through actin polymerization, which is dependent on the Arp2/3 complex and WASP/WAVE proteins, together with the barbed end nucleation factors of mDia and Ena/VASP family proteins and the barbed end-capping proteins of Eps8 (Mattila and Lappalainen, 2008; Jacquemet et al., 2015; Blake and Gallop, 2023). Myosin motor proteins also cooperate in the formation of filopodia and microvilli (Tokuo and Ikebe, 2004; McConnell et al., 2009; Meenderink et al., 2019; Gaeta et al., 2021). The proteins with the inverse Bin-Amphiphysin-Rvs (I-BAR) domain-containing proteins (I-BAR proteins), such as IRSp53, IRTKS, and MIM, connect the actin cytoskeleton to the protrusion membrane (Suetsugu et al., 2006; Scita et al., 2008). These I-BAR proteins can induce protrusion formation directly and are associated with the production of EVs (Hu et al., 2020; Nishimura et al., 2021; de Poret et al., 2022). The I-BAR domain alone can remodel the membrane into protrusions directly, and there is a zone with fewer actin filaments at the tip of the protrusions (Suetsugu et al., 2006; Sudhaharan et al., 2019). The simulation indicates that I-BAR protein recruitment or membrane bending ability can spontaneously induce the pearl/beads-on-a-string-like structure, which can be the precursors of the EVs (Suetsugu et al., 2006; Ravid et al., 2023). The production of phosphatidic acid by the cytoplasmic phospholipase D_2_ enhances protrusion formation, highlighting the involvement of lipid metabolism (Shen et al., 2002). The I-BAR-dependent EVs are enriched in one acyl-chain lysophospholipids, which potentially makes membrane fragile (Nishimura et al., 2021). Therefore, the tip of the membrane, lacking actin filaments, is potentially a fragile structure that may be permissive to fission and serves as the source of EVs.

The physiologically relevant friction force exerted on the protrusions by the flow of the medium plays a key role in the cutting of the tip (Nishimura et al., 2021). Shear stress induced by fluid flow has been shown to trigger vesiculation from the cells cultured under constant agitation (Mohieldin et al., 2021; Nishimura et al., 2021). Several studies have also suggested the possibility that shear force from blood flow in capillaries influences EV production (Hyenne et al., 2019; Verweij et al., 2019). Additionally, the pulling force that is applied to protrusions upon contact with neighbor cells or the substratum is also considered to provide the force for tip scission as well as friction due to cellular contractility or tissue deformation.

The endosomal sorting complex required for transport (ESCRT) machinery is a sequential cytoplasmic protein assembly involved in deforming and cutting lipid membranes. Plasma membrane budding and the release for the human immunodeficiency virus-1 (HIV-1) particles and small ectosomes ARRDC1-mediated microvesicles (ARRMs) are under the control of ESCRT machinery (Nabhan et al., 2012; Votteler and Sundquist, 2013). Furthermore, HIV-1 and Pseudorabies virus utilize IRSp53 for their budding (Yu et al., 2019; Inamdar et al., 2021) (Figure 1D), although, the involvement of ESCRT in cellular protrusions is still not fully understood.

It is unclear how the length of the protrusion can be determined. External cues, including growth factor stimulation, enhance protrusion formation through the activation of cellular signaling proteins, including tyrosine kinases and small GTPases. The above-mentioned actin regulators and I-BAR proteins are all cytoplasmic proteins, lacking transmembrane regions, and can be assembled through multivalent protein interactions downstream of these signaling proteins (Feng et al., 2022; Wan Mohamad Noor et al., 2023).

### 2.2 Transmembrane proteins

Representative transmembrane proteins at protrusions include receptor tyrosine kinases, cell adhesion proteins such as cadherins for cell-cell interactions, and integrins for cell-substratum adhesion proteins with the extracellular matrix (Rabinovitz and Mercurio, 1997; Vasioukhin et al., 2000; Robles et al., 2005). The cell adhesion status, including the area that forms the adhesion contact, greatly affects filopodia formation (Mukherjee et al., 2023). Furthermore, a pentaspan transmembrane glycoprotein, Prominin-1/CD133 (Weigmann et al., 1997) and tetraspan transmembrane proteins, tetraspanin, including CD9 and CD81 (Peñas et al., 2000) are enriched at plasma membrane protrusions. Tetraspanin CD63 is especially enriched in endosome membranes but can also localize to the plasma membrane through endosome fusion with the plasma membrane (Welsh et al., 2024; D’Angelo et al., 2023) (Figure 1E). Endosome-derived EV formation depends on the ESCRT, while plasma-membrane-derived vesicles can serve as an alternative pathway when ESCRT is inhibited (Nishimura et al., 2021). Hyaluronan synthases, including HAS3, are also enriched in protrusions (Rilla et al., 2013). These proteins often serve as marker proteins for EVs. However, the causality of these proteins in the formation of EVs has been enigmatic.

## 3 Protrusions in the inter-cellular material transfer

### 3.1 Filopodia

Filopodia are finger-like protrusions of the cell membrane, having 0.1–0.3 microns in diameter, and with varying lengths. Filopodia can participate in a broad range of cellular processes, including cell migration and adhesion to the extracellular matrix (Arjonen et al., 2011), cue-sensing (Heckman and Plummer, 2013), tissue morphogenesis (Fairchild and Barna, 2014), and pathogen invasion (Chang et al., 2016). Filopodia are well characterized at the leading edge of migrating cells, dendrite and growth cone of neuronal cells.

Emerging studies demonstrating that filopodia transmit signaling molecules were mostly conducted in *Drosophila* (Hsiung et al., 2005; Callejo et al., 2011; Roy et al., 2011; Bilioni et al., 2013; Bischoff et al., 2013; Fereres et al., 2019; Patel et al., 2022; Clements et al., 2024), human cell lines (Stanganello et al., 2015; Mattes et al., 2018; Hu et al., 2020; Brunt et al., 2021; Nishimura et al., 2021; de Poret et al., 2022), and vertebrates such as chick embryos (Sanders et al., 2013), zebrafish (Stanganello et al., 2015; Mattes et al., 2018; Brunt et al., 2021), and mice (Mattes et al., 2018; Brunt et al., 2021; Hall et al., 2021) (Table 1). The maximum recorded length of filopodia in *Drosophila* wing imaginal disc is surprisingly longer than those reported in vertebrates, which are 700 µm and 150 μm, respectively (Ramirez-Weber and Kornberg, 1999; Sanders et al., 2013), implying the variety dependent on experimental models. Filopodia in vertebrates and invertebrates are able to transfer signaling molecules, such as Decapentaplegic (Dpp) (Hsiung et al., 2005; Roy et al., 2011; Fereres et al., 2019), Hedgehog (Hh) (Callejo et al., 2011; Bilioni et al., 2013; Bischoff et al., 2013; Gradilla et al., 2014), Sonic Hedgehog (Shh) (Sanders et al., 2013; Hall et al., 2021; Hall et al., 2024), Notch (de Joussineau et al., 2003; Cohen et al., 2010; Clements et al., 2024), and Wnt (Stanganello et al., 2015; Mattes et al., 2018; Brunt et al., 2021; Hall et al., 2024) to distant cells through physical contact or vesicle release (Table 1). Several of the above-mentioned filopodia and vesicle are dependent on the I-BAR proteins (Table 1). In several studies, plasma-membrane-derived vesicles do not seem to travel through the body fluid or the culture medium. Rather, the tip appears to bind to the recipient cell, after which the tip is excised to form the vesicle. Alternately, if recipient cells are in a close vicinity, the vesicles will travel a very short distance before being immediately captured. These observations suggest that the transfer by plasma-membrane-derived EVs and by filopodia are similar (Figures 1F, G).

**TABLE 1:** Cytonemes, filopodia, and EVs.

The name of the protrusions in the report	Experimental model	Reported length	Core component	Vesicle observed	Vesicle released	Vesicle cargo	Biological function	Literature
Cytonemes	*Drosophila* wing imaginal disc and mouse limb buds	Maximum: 700 µm	Actin	Yes	Yes	Unknown	Unknown	Ramirez-Weber and Kornberg (1999)
Cytonemes	*Drosophila* wing imaginal disc	Average: 20.8 µm Maximum: 80.2 µm	Actin	Unknown	Unknown	Unknown	Dpp signalling	Hsiung et al. (2005)
Filopodia	*Drosophila* notum	Maximum: ∼7 μm	Actin, Rac, SCAR complex	Unknown	Unknown	Unknown	Delta-Notch signaling	Cohen et al. (2010)
Filopodia	*Drosophila* wing imaginal disc	Maximum: 250 µm	Actin, ezrin	Unknown	Unknown	Unknown	Delta-Notch signaling	de Joussineau et al. (2003)
Cytonemes	*Drosophila* wing disc and the abdominal epidermis	Maximum: 70 μm	Actin, SCAR/WAVE, Pico (lamellipodin)	Yes	Yes	Ihog	Hh signaling	Bischoff et al. (2013)
Cytonemes	*Drosophila* wing-disc adult muscle progenitor	Maximum: 15 μm	Actin, Diaphanous	Unknown	Unknown	Unknown	FGF signaling	Patel et al. (2022)
Filopodia	*Drosophila* bristle precursor cells	Average: 7.1 µm	Myosin XV	Unknown	Unknown	Unknown	Notch signaling	Clements et al. (2024)
Cytonemes and the derived EVs	*Drosophila* wing imaginal disc	Unknown	Unknown	Yes	Yes	Hh, Ihog, Dispatched, Dally-like, CD63	Hh signaling	Gradilla et al. (2014)
Filopodia	Chick embryos limb bud mesenchymal cells	Average: 34.27 µm Maximum: 150 µm	Actin, cofilin	Yes	No	SHH	Shh signaling	Sanders et al. (2013)
Filopodia	Zebrafish embryos	Average: 16.6 µm Maximum: 50 µm	Actin, Cdc42, N-Wasp, IRSp53, Myosin X	Unknown	Unknown	Wnt8a	Wnt/β-catenin signaling	Stanganello et al. (2015)
Filopodia and the derived EVs	HEK293	Unknown	MIM, IRSp53, and LysoPE	Yes	Yes	Nectin-2, IRS4, and Rac1	Cell migration	Nishimura et al. (2021)
Filopodia and the derived EVs	Ca9-22	Unknown	IRSp53	Yes	Yes	Unknown	Cancer cell proliferation	Hu et al. (2020)
Filopodia and the derived EVs	HEK293T	Unknown	IRSp53, Arp2/3	Yes	Yes	ITGB1	Unknown	de Poret et al. (2022)
Cytonemes	Zebrafish embryos	Unknown	IRSp53, Cdc42	Unknown	Unknown	Unknown	Wnt/β-catenin signaling	Mattes et al. (2018)
Cytonemes	NIH3T3 and MEFs	Unknown	Myosin X	Yes	No	Unknown	SHH signaling	Hall et al. (2021)
Cytonemes	Mouse neural tubes	Unknown	Actin, myosin Xs	Unknown	Unknown	Unknown	SHH and WNT signaling	Hall et al. (2024)
Cytonemes	PAC2, mouse intestinal telocytes and human gastric cancer cells	Average: 7.9 µm	IRSp53	Unknown	Unknown	Unknown	Wnt/β-catenin signaling	Brunt et al. (2021)

The recipient cells receive these EVs presumably through their surface receptors. Subsequently, the EVs either fuse with the plasma membrane or are endocytosed and then fuse with endosomes to release their content. Although the mechanisms underlying the release of the bioactive cargos from EVs are largely unknown, they are supposed to be common to those for the endosome-derived EVs (Figures 1E–G).

### 3.2 Cytonemes

The potential role of filopodia in intercellular signaling was indicated nearly 25 years ago, when long, slender cellular protrusions emerging from *Drosophila* wing imaginal disc, named “cytonemes,” were shown to orient toward morphogen (Ramirez-Weber and Kornberg, 1999). In the term “cytoneme,” “cyto” refers to the presence of cytoplasmic materials, and “neme” denotes the finger-like appearance, which is the feature of filopodia. Although it is still unclear whether all filopodia are capable of exchanging signals between cells, cytoneme (Hsiung et al., 2005; Mattes et al., 2018), signaling filopodia (Clements et al., 2024), and specialized filopodia (Sanders et al., 2013) are considered to refer the *bona fide* filopodia.

### 3.3 Microvilli

Microvilli and filopodia share a similar molecular architecture, consisting of dense, parallel actin bundles and contain I-BAR proteins and myosin (Figure 1C). Microvilli are often found on the apical surface of epithelial cells, where they provide a larger surface area for nutrient absorption. However, new research suggests a novel role for microvilli as a source of EVs. Studies have shown that the neuroepithelium microvilli in the mouse brain can release prominin-1-containing EVs, which function in tissue differentiation (Marzesco et al., 2005). These EVs appear to preferentially bind to protrusion sites on both epithelial and non-epithelial cells (Weigmann et al., 1997; Marzesco et al., 2005; Karbanová et al., 2008). The shedding of EVs from the distal tips of microvilli has also been reported in various tissues, including *Drosophila* wing imaginal disc epithelium (Hurbain et al., 2022), rat enterocytes (McConnell et al., 2009), and placenta (Davies et al., 2022). Prominin-1 has a cholesterol-binding domain, and the removal of cholesterol has been shown to alter the distribution of prominin at the microvilli (Röper et al., 2000; Marzesco et al., 2009). The depletion of cholesterol from microvilli renders the microvilli unstable and triggers the release of EVs from the barbed end of protrusions (Marzesco et al., 2009). Myosins, known for transporting proteins to the tips of filopodia, have also been implicated in driving the release of EVs from microvilli, such as myosin-1a in the intestinal lumen (McConnell et al., 2009). The involvement of cytoskeletal proteins and BAR protein in the shedding of microvilli EVs is still unknown, and it will be an interesting topic for future research.

### 3.4 Tunneling nanotubes

Tunneling nanotubes (TNTs) were first identified in rat pheochromocytoma PC12 cells as the actin-rich membrane protrusions that connect distant cells and transport membranous vesicles (Rustom et al., 2004). Emerging studies revealed the presence of TNTs in various cell types, including immune cell line (Onfelt et al., 2006; Chinnery et al., 2008; Sowinski et al., 2008; Dupont et al., 2020), epithelial cells (Gurke et al., 2008; Wang et al., 2010; Wang and Gerdes, 2012), neuronal cells (Gousset et al., 2009; Wang X. et al., 2012; Wang and Gerdes, 2012), adenocarcinoma (Wang Z.-G. et al., 2012; Wang and Gerdes, 2012), vascular endothelial cells (Wang and Gerdes, 2012), rat cardiomyocytes (Koyanagi et al., 2005), and myoblast cell (He et al., 2010). The feature of TNTs is different from filopodia or cytoneme, where TNTs are open-ended structures that transport the cargo along the long tubules to the distant cells without exocytosis and touching the substratum (Rustom et al., 2004; Abounit and Zurzolo, 2012; Dupont et al., 2018) (Figure 1H). The interconnected tubes are transient and sensitive to stresses such as light exposure and frictional force (Rustom et al., 2004). The reported lengths of TNTs vary, from a few micrometers to a few hundred micrometers, and the maximum recorded length is 300 µm (Rustom et al., 2004; Chinnery et al., 2008; He et al., 2010; Wang Z.-G. et al., 2012).

Some studies reported that there are two subtypes of TNTs, which are constituted of both actin and microtubules or actin alone, with different diameters (Onfelt et al., 2006; Wang X. et al., 2012; Wang Z.-G. et al., 2012). IRSp53 and Eps8 are involved in the TNT formation (Henderson et al., 2023). Driven by actin polymerization, TNTs extend from a cell, and the distal tip fuses with the plasma membrane of another cell. The biological roles of TNTs include transporting organelle in between cells (Onfelt et al., 2006; Gurke et al., 2008; Wang and Gerdes, 2015), transferring receptor complexes to mediate cell immune responses (Chinnery et al., 2008), and HIV-1 transmission (Sowinski et al., 2008; Dupont et al., 2020). The mechanism of transportation was reported to be facilitated by Myosin Va, a protein involved in organelle transport (Rustom et al., 2004).

### 3.5 Viral budding

One of the prerequisites for the budding of viral particles, such as those of HIV-1, is the formation of membrane curvature. The membrane budding was thought to be solely dependent on the virus Gag protein, but studies revealed that the I-BAR protein, IRSp53, plays a crucial role in the assembly and budding of the viral particles (Thomas et al., 2015; Inamdar et al., 2021). Both Gag and IRSp53 can interact with phosphatidylinositol 4,5-bisphosphate (PIP_2_) in the inner leaflet of plasma membrane (Prévost et al., 2015; Favard et al., 2019; Sengupta et al., 2019), and the activation of small GTPase, Rac1, at the IRSp53-Gag localized membrane drives the viral particle release (Thomas et al., 2015). The involvement of actin cytoskeleton in the virus budding was also reported, where the IRSp53 recruits WAVE2 and Arp2/3 for actin polymerization in the budding site (Thomas et al., 2015). A recent finding in neuronal cells reported that IRSp53 can interact with Arc, which is an intrinsic protein that has similarity with retroviral Gag (Alicia et al., 2024). IRSp53 can facilitate the oligomerization of Arc into capsid at the membrane protrusion site and release EVs that contain the Arc capsid and mRNA (Alicia et al., 2024). Other than IRSp53, Gag, and Arc, the budding and release of viral particles were dependent on the ESCRT machinery (Votteler and Sundquist, 2013). Nevertheless, the interplay between these proteins in the membrane site for virus budding is still yet to be fully discovered.

### 3.6 Proplatelet protrusions from megakaryocytes

Platelets and microvesicles/ectosomes have similar origins, as both are generated through the scission of proplatelets, which are the pseudopod protrusions of megakaryocytes (Behnke, 1968; Machlus and Italiano, 2013). Furthermore, platelets were among the first to demonstrate secretion of EVs. The activated platelets have abundant protrusions on their surface (Sorrentino et al., 2021; Zheng et al., 2021), and the first discovery of EVs arose from particles derived from the platelets, known as “platelet dust,” where initially perceived as cellular waste (Wolf, 1967). The later research revealed that the formation of EVs from most cell types was not always a random process of excretion of waste but actively driven by intricate cellular mechanisms (Raposo and Stoorvogel, 2013; van Niel et al., 2018).

Megakaryocytes are mainly present in bone marrow and are responsible for platelet biogenesis (Ogawa, 1993; Morita et al., 2011). The plasma membrane of megakaryocytes is expandable to 10-fold to serve as the reservoir for cytoskeleton proteins and membrane lipids to generate thousands of platelets. The scission of proplatelets, i.e., the generation of platelets, also relies on shear stress (Ito et al., 2018), which might be reminiscent of the filopodia-derived vesicles.

In proplatelets shedding, the microtubules and actin filaments play crucial roles (Patel et al., 2005; Thon et al., 2010; Machlus and Italiano, 2013). The polymerization of microtubules powers the elongation of proplatelets in cooperation with a microtubule minus end-associated protein, dynein (Lecine et al., 2000; Patel et al., 2005; Machlus et al., 2019). The lack of functional tubulin affects human platelet production as the tubulin mutation was identified in the patient with macrothrombocytopenia (Kunishima et al., 2009).

### 3.7 Cilia

There are two types of cilia, called motile cilia and non-motile cilia. Motile cilia are hair-like protrusions on the surface of epithelial cells underlying the respiratory tract, oviduct, and brain ventricular system, whereas non-motile cilia, such as primary cilium, is the solitary sensory organelle projected from the apical surface of differentiated, non-dividing-cells. Motile cilia facilitate the transport of substances along a passage through wave-like beating motion (Zhou and Roy, 2015), whereas primary cilium has abundant receptors at the tip, and acts as the antenna that transmits signals between cells (Anvarian et al., 2019). Both the motile-cilia and primary cilia on epithelial cells have been evidenced to release EVs from the distal tips (Dubreuil et al., 2007; Kesimer et al., 2009) as well as from the base of the protrusions (Wang et al., 2021).

Contrary to filopodia and microvilli, the core of cilia is composed of microtubule filaments. Therefore, cilia are not generally considered to be similar to filopodia. However, there are several molecular similarities in the EV release. The mechanism of EV secretion from cilia involves the cooperation of actin and myosin components (Nager et al., 2017; Phua et al., 2017). EV secretion from cilia can be triggered by the actin regulatory protein, drebrin, myosin 6 (Nager et al., 2017), as well as phospholipid PIP_2_ (Phua et al., 2017). Similar to the EVs from microvilli, the EVs released from the neuroepithelial primary cilium contain prominin-1 (Dubreuil et al., 2007), and the shedding of EVs from cilia serves in transmitting Hh signaling (Nager et al., 2017).

## 4 Membrane protrusions and EVs in cancer cells

Studies have shown that the abundance of membrane protrusive structures, such as filopodia, is highly related to cancer progression and metastasis. In cancer, increased filopodia density often correlates with cell migration and metastasis of cancer cells (Jacquemet et al., 2015; Jacquemet et al., 2017). Several studies have shown that cancer cells secrete more EVs as observed in cancer-patient samples in comparison with healthy patients-derived samples (Khan et al., 2012; Puhka et al., 2017), and *in vitro* comparison of cancer cells with their non-cancer cellular counterparts (Riches et al., 2014).

Furthermore, EVs from cancer cells exhibit a unique profile compared to those of normal cells (György et al., 2011). Likewise, commonly used chemotherapeutic regimen affects EV distribution and function (Tzoran et al., 2015; Aharon et al., 2017) by enhancing the release of EVs with pro-metastatic (Keklikoglou et al., 2019; Wills et al., 2021), pro-chemotherapeutic resistance (Wang et al., 2019), and pro-angiogenic (Zarfati et al., 2019) abilities. The EVs derived from cancer cell membrane protrusions have also been reported to promote tumorigenesis and metastasis (Muralidharan-Chari et al., 2009; Härkönen et al., 2019; Hu et al., 2020).

Although these studies have not completely examined the protrusions as their EV sources, these findings strongly suggest the importance of membrane protrusions as a platform for the generation of cancer cell-derived EVs, which will be useful as prognostic markers or therapeutic targets.

## 5 Conclusion and perspective

The plasma membrane serves as a versatile platform that can generate various forms of protrusive structures. These protrusions play a crucial role in the generation of EVs that are important for cell-to-cell communication. Given the diverse varieties of protrusions and the understanding of their molecular mechanisms, protrusion-derived EVs can be a powerful tool in disease prognosis, especially in cancer. By elucidating the components involved in EV secretion from protrusive structures, it is possible to utilize a protrusive structure as a personalized system for EV secretion in cancer therapy.
